# Neural substrates underlying rhythmic coupling of female reproductive and thermoregulatory circuits 

**DOI:** 10.3389/fphys.2023.1254287

**Published:** 2023-09-11

**Authors:** Azure D. Grant, Lance J. Kriegsfeld

**Affiliations:** ^1^ Levels Health Inc, New York, NY, United States; ^2^ Department of Psychology, University of California, Berkeley, CA, United States; ^3^ The Helen Wills Neuroscience Institute, University of California, Berkeley, CA, United States; ^4^ Department of Integrative Biology, University of California, Berkeley, CA, United States; ^5^ Graduate Group in Endocrinology, University of California, Berkeley, CA, United States

**Keywords:** HPG, TIDA, ventral tegmental area, biological rhythms, coupled oscillators, network physiology

## Abstract

Coordinated fluctuations in female reproductive physiology and thermoregulatory output have been reported for over a century. These changes occur rhythmically at the hourly (ultradian), daily (circadian), and multi-day (ovulatory) timescales, are critical for reproductive function, and have led to the use of temperature patterns as a proxy for female reproductive state. The mechanisms underlying coupling between reproductive and thermoregulatory systems are not fully established, hindering the expansion of inferences that body temperature can provide about female reproductive status. At present, numerous digital tools rely on temperature to infer the timing of ovulation and additional applications (e.g., monitoring ovulatory irregularities and progression of puberty, pregnancy, and menopause are developed based on the assumption that reproductive-thermoregulatory coupling occurs across timescales and life stages. However, without clear understanding of the mechanisms and degree of coupling among the neural substrates regulating temperature and the reproductive axis, whether such approaches will bear fruit in particular domains is uncertain. In this overview, we present evidence supporting broad coupling among the central circuits governing reproduction, thermoregulation, and broader systemic physiology, focusing on timing at ultradian frequencies. Future work characterizing the dynamics of reproductive-thermoregulatory coupling across the lifespan, and of conditions that may decouple these circuits (e.g., circadian disruption, metabolic disease) and compromise female reproductive health, will aid in the development of strategies for early detection of reproductive irregularities and monitoring the efficacy of fertility treatments.

## Introduction

Female endocrine and thermoregulatory outputs exhibit coordinated rhythms at within-a-day (ultradian; UR) (Sanchez-Alavez et al., 2011; Smarr et al., 2017; Goh et al., 2019; Grant et al., 2020), daily (circadian; CR) (Kerdelhué et al., 2002), and ovulatory (OR) timescales (van de Velde, 1926; de Mouzon et al., 1984). This broad pattern of rhythmic harmony suggests that neuroendocrine physiology operates as a network of coupled oscillators across systems and timescales (Brandenberger et al., 1987; Van Cauter, 1990; Shannahoff-Khalsa et al., 1996; Bourguignon and Storch, 2017; Grant et al., 2018; Goh et al., 2019). Although numerous commercial tools have been developed based on this assumption, whether rhythmic patterns are generalizable across individuals in real-world settings, and stable across life stages, is unknown. Most of the work to date has focused on the utility of temperature patterns in ovulatory cycle and fertility tracking, with substantial success in both domains (Goeckenjan et al., 2020; Grant et al., 2020; Alzueta et al., 2022). More recent work has extended the use of rhythmic temperature patterns to successful detection of pregnancy and early prediction of pregnancy complications (Smarr et al., 2016b; Webster and Smarr, 2020; Grant and Smarr, 2022). The use of temperature fluctuations to monitor the menopausal transition, in the presence or absence of hormone replacement therapy, is more limited but represents a topic of broad importance for female health during older age (van de Velde, 1905; Murphy and Campbell, 2007; Grant et al., 2020). The present overview describes direct and indirect evidence of coupling among the female reproductive axis, thermoregulatory circuits, and system-wide physiology, and argues for the utility of non-invasive temperature monitoring to characterize normative rhythmic patterns across the lifespan, detect deviations from typical trajectories, and monitor the impact of reproductive clinical treatments.

The study of temporal coupling among body temperature and reproductive state dates to over a century ago with the Dutch gynecologist and author, Theodor Van de Velde. Van de Velde documented the use of daily oral temperature to monitor the ovulatory cycle, pregnancy, and the transition to menopause (van de Velde, 1905; van de Velde, 1926). The work revealed a temperature rise during the luteal phase in premenopausal women, the absence of this pattern in menopause, and a sustained temperature rise in early pregnancy. These changes have since been ascribed to the temperature-lowering effect of estrogen, and the temperature-elevating effect of progesterone (Buxton and Atkinson, 1948; Silva and Boulant, 1986; Charkoudian and Johnson, 2000; Stachenfeld et al., 2000; Mittelman-Smith et al., 2012; Zhang et al., 2021). Although this coupling between reproductive state and thermoregulation is often observed and utilized on the order of weeks to months, more recent work has revealed coupling at ultradian timescales (Shannahoff-Khalsa et al., 1996; Grant et al., 2018; 2020; Goh et al., 2019). Together, findings over the last several decades in reproductive neuroendocrinology, thermoregulation, and biological rhythms revealed that temperature and reproductive output exhibit multi-scale rhythms that drive reproductive function (e.g., ovulation), and that these rhythms may be coupled on the order of hours, consistent with the ability of temperature to report pulsatile hormone secretion. Monitoring such coupling through non-invasive metrics like temperature could not only provide regular readouts of reproductive state, but also enable prediction of future reproductive state and potential fertility issues without hormone sampling (e.g. (Smarr et al., 2016b; Erickson et al., 2023)). As a result, the study of continuous temperature and hormones in females represents an important opportunity to further understand endocrine and metabolic network dynamics, with rapid, real-world translational applications (Shannahoff-Khalsa et al., 1996; Bashan et al., 2012; Bartsch et al., 2015; Webster et al., 2015; Grant et al., 2018; Goodale et al., 2019; Grant et al., 2020; Webster and Smarr, 2020).

If patterns of hormonal change over time (URs, CRs, and ORs) are strongly coupled to patterns in easy-to-measure outputs such as temperature, then wearable sensors could be broadly applied to provide information about reproductive state. Potential applications include personalized prediction or detection of pubertal onset and progression, ovulation, pregnancy and potential complications, labor onset, sub- and infertility, reproductive aging, and guidance/monitoring during hormone replacement (Farris, 1947; Buxton and Atkinson, 1948; Cohen et al., 1976; Grant et al., 2020; Webster and Smarr, 2020; Grant et al., 2021; Grant and Erickson, 2022; Grant and Smarr, 2022). Of these potential applications, once-daily ovulatory cycle monitoring has been realized at scale (Bull et al., 2019), with daily oral or skin temperature commonly used as an approximate marker of ovulation for the purposes of family planning (van de Velde, 1905; van de Velde, 1926; Buxton and Atkinson, 1948; de Mouzon et al., 1984; Bull et al., 2019; Maijala et al., 2019).

**FIGURE 1:**
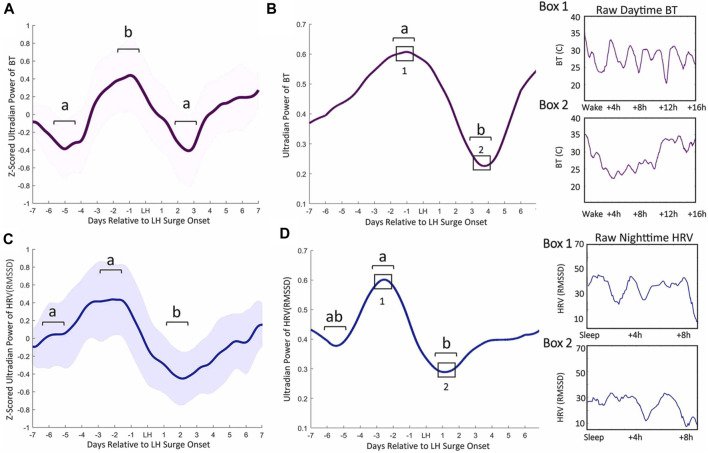
Ultradian power of distal BT and heart rate variability (HRV) anticipates LH surge onset. Mean BT **(A)** and root mean squared standard deviation (RMSSD, a measure of HRV) **(C)** ultradian power (z-scored) ± standard deviation for cycles within 1 week of the LH surge in women. BT UR power peaks exhibit an inflection point 5.82 (±1.82) days prior to LH onset, a peak a mean of 2.58 (±1.89) days before LH onset and a subsequent trough 2.6 (±1.02) days after surge onset (Grant et al., 2020) **(A)**. Ultradian HRV power inflects an average of 5.82 (±1.53) nights prior to LH surge onset, exhibits a subsequent peak 2.58 (±1.59) days prior to the surge onset and a trough 2.11 (±1.27) days after surge onset **(C)**. Representative individual example of raw BT ultradian power within 1 week of LH surge onset **(B)** and raw HRV one week before the LH surge **(D)**. Black squares in B and D correspond to Boxes 1 and 2 that show linear waking BT and HRV from which ultradian power in B and D were generated. (from (Grant et al., 2020)).

Recent work by our group and others revealed that it is possible to anticipate ovulation through continuous monitoring of ultradian rhythms in body temperature that presumably mirror underlying changes in estrogen and progesterone release patterns across the ovulatory cycle (Prendergast et al., 2012; Smarr et al., 2017; Grant et al., 2020) (Figure 1). Although such findings are promising, it cannot be assumed that the coupled oscillator hypothesis is reliable across time scales, individuals, and stages of reproductive life. For example, coupling observed at the timescale on the order of months, such as that of basal body temperature and progesterone during the menstrual cycle, does not imply that coupling between temperature and sex steroid hormones occurs at circadian or ultradian timescales. Additionally, in modern society, individuals are subject to rhythmic disruption in many forms (e.g., light at night, blue-light-emitting devices) and may exhibit weaker relationships between hormones and body temperature as a result. Finally, physiological states resulting from exogenous hormone administration such as birth control and post-menopausal hormone replacement therapy, might perturb coupling and negatively impact the ability of temperature to report hormonal state. The stronger the coupling between thermoregulation and reproduction, the more likely that coupling occurs across timescales and is resilient to disruption. To begin to consider these possibilities, it is instructive to review evidence for central coupling among reproductive and thermoregulatory loci, as well as the origins of reproductive and thermoregulatory pulsatility. Our hope is that this overview will stimulate future studies evaluating the nature of and mechanisms underlying putative coupling pathways to help guide consideration of ideal, non-invasive proxy metrics for reproductive health across the female lifespan.

### Hypothalamic organization of reproduction

Multiple hypothalamic subregions coordinate the release of reproductive neuropeptides and hormones (Plant, 2015), including kisspeptin within the arcuate (ARC) and anteroventral paraventricular (AVPV) nuclei, gonadotropin releasing hormone (GnRH) within the preoptic area (POA) and anterior hypothalamus, and RFamide-related peptide 3 (the mammalian ortholog of avian gonadotropin-inhibitory hormone) within the dorsomedial nucleus of the hypothalamus (Ubuka et al., 2018; Angelopoulou et al., 2019). These populations integrate with the central clock in the suprachiasmatic nucleus (SCN) and extrahypothalamic regions to generate an exquisitely coordinated circadian-, ovulatory phase-, pulse-, and environmentally-modulated GnRH signal (reviewed in (Moeller et al., 2022; Piet, 2023)). GnRH is released to the portal vasculature of the anterior pituitary where it triggers the release of the gonadotropins, luteinizing hormone (LH) and follicle stimulating hormone (FSH). LH and FSH act on the gonads to stimulate the synthesis and release of sex steroids and gamete maturation, respectively. Sex steroids and gonadotropins provide feedback both locally and at the levels of the pituitary and hypothalamus to regulate hypothalamo-pituitary-gonadal (HPG) axis activity (Plant, 2015; Herbison, 2018).

URs are integral to the functioning of the HPG axis and across the ovulatory cycle (Backstrom et al., 1982; Herbison, 2018). Much of the data on GnRH pulsatile release has been garnered from ewes as GnRH can be more easily sampled in this large ungulate relative to rodents. In ewes, each GnRH UR or “pulse” consists of rapid rise, sustained elevation with a 4 or 5 min variable plateau, and a precipitous ∼3 min decline (Moenter et al., 1991; Moenter et al., 1992; Goodman et al., 1995). This pattern repeats with stereotyped frequency modulation across the ovulatory cycle. The pulsatile signal generated by these complex interactions propagates down the HPG axis, resulting in URs of LH, estrogens, progesterone, and testosterone (Backstrom et al., 1982; Albertsson-Wikland et al., 1997; Grant et al., 2018). This patterning is required for normal pituitary gonadotropin secretion (Thompson and Kaiser, 2014; Zavala et al., 2019), and its disruption may contribute to polycystic ovarian syndrome (PCOS) (Chaudhari et al., 2018; Coutinho and Kauffman, 2019; Hunjan and Abbara, 2019), hypothalamic amenorrhea (Meczekalski et al., 2014; Fourman and Fazeli, 2015), hypogonadotropic hypogonadism (Belchetz et al., 1978; Gronier et al., 2014; Hao et al., 2021), and menopause (Hunjan and Abbara, 2019).

In women, HPG axis pulse frequency increases from about once per 1–2 h, to slightly more than once per hour, across the pre-ovulatory or follicular phase of the cycle (Backstrom et al., 1982; Grant et al., 2018). This change is coincident with rising levels of estradiol that peak prior to ovulation, and low concentrations of progesterone. In spontaneously-ovulating rodents, sufficiently elevated estradiol integrates with SCN vasopressin (AVP)-ergic circadian signaling at kisspeptin neurons of the AVPV to stimulate the LH surge that initiates ovulation (Moeller et al., 2022; Moralia et al., 2022; Tonsfeldt et al., 2022; Piet, 2023). In addition, SCN vasoactive-intestinal polypeptide (VIP)-ergic projections to the POA also contribute to the LH surge via direct communication to GnRH cells (Moeller et al., 2022; Moralia et al., 2022; Tonsfeldt et al., 2022; Piet, 2023). Although numerous lines of evidence point to the negative impact of circadian disruption on ovulation in women, the SCN does not appear to be necessary for the LH surge in women and higher primates (reviewed in (Moeller et al., 2022)). Despite a role for circadian coordination of ovulation timing, and reports of post-ovulatory reductions in circadian amplitude of body-temperature CRs (Webster and Smarr, 2020), CR stability does not appear to change stereotypically across the ovulatory cycle in women (Grant et al., 2020). Following the LH surge and ovulation, pulsatility slows to one pulse per 3 or 4 h (Moenter et al., 1991; Goodman and Inskeep, 2015; Grant et al., 2018). This post-ovulatory, luteal phase of the cycle is associated with elevated progesterone and a relatively small elevation in estradiol. In the absence of pregnancy, progesterone declines in the latter portion of the luteal phase, leading to the onset of menses and the eventual beginning of a new cycle.

Ultradian rhythmicity in the HPG axis appears to originate, at least in part, from a kisspeptin pulse generator located in the ARC (Lehman et al., 2010; Herbison, 2018), a region of the hypothalamus also implicated as a broader source of hormonal and behavioral URs (Prendergast and Zucker, 2016). The key to understanding GnRH pulsatility came with the identification of triple-phenotype neurons in the ewe ARC that express kisspeptin/Neurokinin/Dynorphin, or KNDy, neurons (Goodman et al., 2007; Nestor et al., 2023). With reciprocal connectivity among ARC kisspeptin neurons, it appears that neurokinin (NKB) communication initiates a pulse whereas dynorphin suppresses kisspeptin neuronal activity to terminate a pulse, and that these rhythms neuronal activity stimulate GnRH cells via kisspeptin release (Lehman et al., 2010; Lehman et al., 2019; Nestor et al., 2023). One recent finding suggests a potential modification to this hypothesis wherein pulses originate from glutamate-AMPA mediated spontaneous synchronization among kisspeptin neurons. Under this proposed model, dynorphin-kappa opioid tone gates the initiation of synchronization to drive pulsatility and facilitate kisspeptin cell synchrony (Han et al., 2023).

Arcuate KNDy neurons appear to be common across species, and were recently shown to form close, non-synaptic appositions to GnRH cell “dendrons”. These unique structures are positioned at the median eminence (ME), resemble both an axon and dendrite, and release kisspeptin to control GnRH activity (Liu et al., 2021). In mice, KNDy neurons exhibit synchronized bursting correlated with pulsatile LH secretion (Clarkson et al., 2017). Likewise, optogenetic stimulation of KNDy cells results in corresponding LH pulses (Han et al., 2015) and their selective suppression inhibits LH pulses (Clarkson et al., 2017). Although these circuits are frequently studied in rodents and ewes, kisspeptin neurons in the rostral POA, infundibular nucleus, and ARC (Hrabovszky et al., 2010) potentially take on the role of pulse generation in humans, although more data are needed to reveal the neurochemical mechanisms of pulse generation in women (Jayasena et al., 2009; Chan et al., 2012; Skrapits et al., 2015; Lehman et al., 2019). Additional mechanisms contribute to the *in vivo* oscillatory dynamics of GnRH neurons, including extra-hypothalamic input (Soper and Weick, 1980). Finally, cultured adult or embryonic GnRH neurons exhibit synchronized pulsatility, suggesting a contribution of intrinsic pulse-generation by these cells (Weiner et al., 1992; Terasawa et al., 1999; Duittoz and Batailler, 2000; Funabashi et al., 2000; Moore et al., 2002; Gore et al., 2004). Together, these observations strongly support kisspeptin-cell-mediated ultradian stimulation of the GnRH system with contributions from extrahypothalamic loci and intrinsic rhythm generation. Precisely how synchronicity is maintained within the pulse generator, as well as mechanisms behind the frequency modulation of pulsatility observed across the ovulatory cycle, remain to be fully understood (Czieselsky et al., 2016; Herbison, 2018).

### Hypothalamic organization of thermoregulation

Body temperature (BT) is a non-stationary signal that is influenced by a variety of temporal, endocrine, autonomic, behavioral, and species-specific factors. Mammalian BT exhibits URs, CRs, and ORs; as well as distinct rhythmic structures at different locations on the body (Krauchi and Wirz-Justice, 1994; Krauchi et al., 2014; Grant et al., 2018; Grant et al., 2021). Although each part of the body comprises a “micro-climate”, BT is traditionally divided into the skin or “shell”, and the “core” or interior of the body (Childs, 2018; Romanovsky, 2018). During the active phase, core temperature is elevated relative to the shell (Krauchi and Wirz-Justice, 1994; Krauchi et al., 2014). During the inactive phase, vasodilation sends blood from the core to the shell, thereby reducing core temperature and heating the shell through heat dissipation (Charkoudian et al., 2017; Tan and Knight, 2018). In larger mammals, including humans, the gradient from shell to core is more exaggerated than in smaller animals, such as mice and rats (Weiss et al., 2017; van der Vinne et al., 2020; Reid et al., 2021), suggesting that findings from rodent core and shell may not translate directly to human core and shell temperature (Krauchi et al., 2014; Webster and Smarr, 2020). For simplicity, the present discussion will be limited to central regulation of core temperature (CBT) (Batinga et al., 2015; Maijala et al., 2019; Grant et al., 2020). For reviews on behavioral and environmental influences on body temperature, see (Charkoudian and Stachenfeld, 2011; Morrison and Nakamura, 2011; Krauchi et al., 2014; Webster and Smarr, 2020).

Thermoregulation is centrally controlled by neural populations within the POA (Tan and Knight, 2018). Activity across the rat POA, which includes both reproductive and thermoregulatory cell populations, is pulsatile during estrus and diestrus (Pardey-Borrero et al., 1985), with some POA neurons exhibiting ultradian bouts associated with REM sleep and sinusoidal ultradian waking bouts (Miyamoto et al., 2012). “Warm-sensitive” neurons, named due to their activation at high temperatures via transient receptor potential, or TRP, channels (Wang and Siemens, 2015) make up ∼30% of the POA (Boulant and Dean, 1986; Tan et al., 2016). Warm-sensitive neurons appear to reciprocally inhibit or override a much smaller population of “cold-sensitive” neurons, which may occupy ∼5–10% of the POA (Boulant and Dean, 1986; Nakamura and Morrison, 2008; Tan et al., 2016) and extend into the dorsomedial hypothalamus (DMH) (Zhao et al., 2017). Additionally, GABAergic interneurons within the POA serve to inhibit warm-sensitive neurons in response to cold stimuli (Morrison et al., 2008). Control of heat dissipation is accomplished via the impact of these neurons on autonomic tone at cutaneous arterioles, and heat generation is accomplished via impact on brown adipose tissue (BAT) and skeletal and erector pili muscle activity. Environmental feedback to thermoregulatory populations comes from sensory neurons in the trigeminal and dorsal root ganglia that sample temperature from specific tissues in the abdomen, spine, skin, and within the brain itself (Wit and Wang, 1968; Boulant and Hardy, 1974; Silva and Boulant, 1986; Vriens et al., 2014; Tan and Knight, 2018).

### Interaction among thermoregulatory and reproductive circuits

In animal models, the association between increased core and skin temperature measurements have given rise to the term “in heat” for sexually receptive females (Marrone et al., 1976). Acutely, high levels of estradiol around the time of ovulation promote peripheral vasodilation, followed by high progesterone concentrations during the luteal phase leading to peripheral vasoconstriction (Charkoudian et al., 2017). Accordingly, estradiol lowers core and skin temperature in females (Williams et al., 2010; Mittelman-Smith et al., 2012; Rance et al., 2013), and ovariectomized mice (in which the dominant estradiol source is removed) exhibit a sustained increase in tail temperature that can be reversed with estradiol treatment (Ding et al., 2013). In males, testosterone acutely raises muscle temperature while lowering adipose temperature, but this hormone can also be aromatized to estradiol, thereby affecting body temperature through similar mechanisms as estradiol in females (Mauvais-Jarvis, 2011; Clarke et al., 2012). Conversely, progesterone, either alone or combined with estradiol, raises body temperature. In rodents, the pre-ovulatory spike in estradiol and progesterone on estrous days is associated with high CBT (Sanchez-Alavez et al., 2010; Szawka et al., 2010; Prendergast et al., 2012; Smarr et al., 2017). This phenomenon has also been observed in humans following ovulation (Maijala et al., 2019; Grant et al., 2020). Additionally, recent work has identified various patterns of pre-ovulatory temperature depression followed by a peri-ovulatory temperature rise, although confounding factors (e.g., assuming LH surges directly report ovulation) limit the interpretation of such findings (Berglund Scherwitzl et al., 2015; Shilaih et al., 2018; Goodale et al., 2019; Kleinschmidt et al., 2019). Our recent work identified consistent increases in peripheral temperature ultradian amplitude and frequency just prior to the onset of the LH surge in women (Grant et al., 2020) (Figure 1), mirroring changes in LH pulse frequency across the menstrual cycle previously reported (Backstrom et al., 1982). As described below, changes in thermogenesis associated with sex steroids throughout the ovulatory cycle likely occur through co-influence on hypothalamic neurons that regulate pulsatile release of GnRH as well as body temperature, and through direct synaptic coupling (Mittelman-Smith et al., 2012; Rance et al., 2013).

**FIGURE 2:**
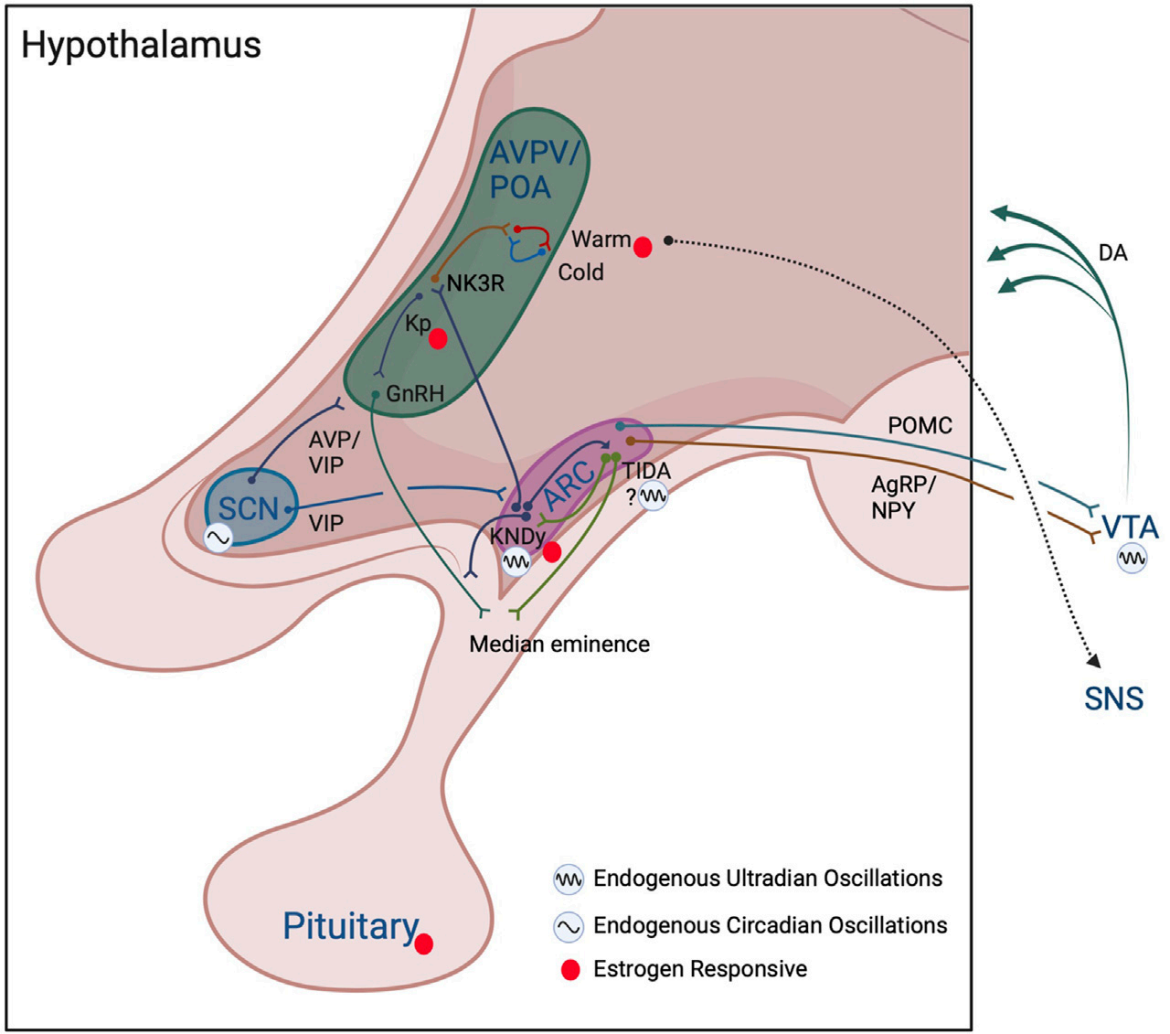
Model of interactions among reproductive and thermoregulatory output in ultradian rhythm generation. Hypothalamic and subcortical structures proposed to enable ultradian coupling of reproductive and thermoregulatory outputs. The KNDy ARC pulse generator conveys a pulsatile GnRH signal to the anterior pituitary, stimulating pulsatile release of LH and FSH. KNDy input to TIDA neurons may act to generate ultradian patterns of dopamine release into the pituitary to temporally influence prolactin secretion. Whether TIDA neurons are capable of intrinsic ultradian rhythmic generation remains to be determined. The KNDy UR network influences warm- (and potentially cold-) thermosensitive-neurons through NK3R glutamatergic POA neurons. The SCN and the dopaminergic ultradian oscillator of the VTA may further impose both time of day (SCN) and time of cycle (VTA via NAcc *not shown*) regulation of ultradian rhythms in body temperature. These actions may further be achieved through influence on the ARC pulse generator, POA/AVPV kisspeptin and GnRH populations, impact of ARC POMC and NPY/AgRP neuronal communication, and VTA projections to more diverse hypothalamic subregions. Likewise, feedback from estrogens throughout the cycle likely acts directly on kisspeptin cells in the AVPV/ARC, on warm-sensitive neurons in the POA, and at the level of the pituitary to further regulate the pattern of URs in temperature. Colors of neuronal projections for clarity only. See text for further details. Created with BioRender.com.

First, a distinct population of non-thermosensitive neurokinin 3 receptor (NK3R) -expressing neurons in the POA are modulated by estrogen-responsive ARC KNDy neurons that release neurokinin (Mittelman-Smith et al., 2012; Mittelman-Smith et al., 2015; Padilla et al., 2018; Krajewski-Hall et al., 2019) (Figure 2). These NK3R-positive neurons are glutamatergic and reduce core temperature following local injections of the NK3R receptor agonist, senktide, or following ablation of these cells, providing a pathway for the changing impact of estrogen on body temperature throughout the ovulatory cycle (Mittelman-Smith et al., 2015; Krajewski-Hall et al., 2019). These cells are not impacted by temperature-sensitive neurons in skin or viscera, and are not warm-responsive POA neurons, pointing to KNDy cells in regulating ultradian temperature patterning indirectly via NK3R-expressing cells in the POA (Krajewski-Hall et al., 2019). In accord with this reasoning, female *Kiss1r* (the gene for the kisspeptin receptor) knockout mice exhibit lower amplitude circadian rhythms in body temperature, likely due to reduction in the frequency of active-phase ultradian temperature events that would normally boost circadian amplitude (Kavanagh et al., 2022). These effects appear to be both centrally and peripherally mediated as mice lacking *Kiss1r* in brown adipose tissue display increased CBT (Tolson et al., 2020). The impact of female reproductive state on BAT is further regulated indirectly via estrogen-responsive sympathetic nervous system (SNS) outflow originating from the ventromedial hypothalamus and the POA (Figure 2) (reviewed in (Zhang et al., 2021)).

In addition to KNDy regulation of temperature via NK3R POA cells, GnRH can excite warm-sensitive neurons in rats, potentially lowering body temperature in an ultradian fashion (Inagaki et al., 1985). To our knowledge, GnRH receptors have not been localized to warm-sensitive neurons and effects of GnRH are likely indirect. Additionally, hot flashes are maintained in postmenopausal women with Kallmann syndrome, a disorder in which GnRH neurons fail to differentiate and migrate from the olfactory mucosa to the hypothalamus, suggesting contributions of GnRH to temperature-sensitive neurons are likely negligible under typical physiological circumstances (Gambone et al., 1984; Rance et al., 2013). Potentially further contributing to ovulatory cycle changes in rhythmic temperature around the time of the LH surge, progesterone largely inhibits warm-sensitive neurons in slice (Tsai et al., 1988; Tsai et al., 1992). Additionally, prostaglandin E2, which is released in response to progesterone (and, canonically, in sickness) generally increases temperature, likely by binding to EP3 receptors on GABAergic neurons in the POA that subsequently communicate to warm-sensitive neurons (Morrison and Nakamura, 2011; Rusyniak et al., 2011).

In addition to these central mechanisms of control, sex steroid hormones feed back to the brain to influence temperature, with about half of warm-sensitive POA neurons affected by estrogens or testosterone (Silva and Boulant, 1986; Zhang et al., 2021). These findings suggest that periodic release of sex steroids could further reinforce ultradian temperature patterns. As might be expected, in ovariectomized animals, the pattern of temperature (Kobayashi et al., 2000; Opas et al., 2004) and the activity of temperature-sensitive cells in the POA is both reduced and disordered (Wang et al., 2014). Conversely, their pattern of activity can be rescued, in part, by phytoestrogen-rich food (e.g., black cohosh) and by direct estradiol replacement (Opas et al., 2004; Hui et al., 2012). Notably, although temperature level is commonly reduced by these interventions, effects on ultradian temperature patterning are more variable, suggesting that tonic administration of estrogens can impact temperature setpoint and ultradian patterning differentially (Opas et al., 2004; Grant et al., 2022). It is likely that temporal patterns of sex-steroid receptor expression are altered at hormone-responsive thermoregulatory loci, leading to changes in ultradian patterning, a possibility requiring further investigation. Together, the activity profile and reciprocal connections among thermoregulatory neurons in the POA, reproductive circuits in the ARC, and sex steroids, suggest that reproductive-thermoregulatory coupling is both centrally generated and peripherally reinforced through feedback to the brain (Figure 2).

### Modulation of URs by phase of the ovulatory cycle: A potential role for the dopaminergic ultradian oscillator, TIDA neurons, and the arcuate pulse generator

In 1980, Soper and Weick suggested that an extrahypothalamic region, and the ARC, may provide two independent mechanisms for the generation of LH pulsatility in ovariectomized rats (Soper and Weick, 1980). Thus, an additional locus may contribute to UR generation across the ARC pulse generator and POA. Twenty five years after the findings by Soper and Weick, the ventral tegmental area (VTA) was compellingly presented as an tunable regulator of URs in dopamine, behavior, and temperature (Blum et al., 2014; Bourguignon and Storch, 2017). This system, dubbed the “Dopamine Ultradian Oscillator”, or DUO, may be responsible for reinforcement of URs and modulation of UR periodicity across the ovulatory cycle. The VTA exhibits URs in dopamine that are tightly correlated to URs in CBT and locomotor activity (Blum et al., 2014; Bourguignon and Storch, 2017). Additionally, endogenous and pharmacological increases in dopamine lengthen UR period, whereas endogenous and pharmacological decreases in dopamine shorten UR period (Blum et al., 2014; Bourguignon and Storch, 2017). Subsequent experiments established that the VTA is a key area for generation and coordination of URs across systems, including motivation, locomotor activity, feeding, and hippocampal activity (Blum et al., 2014; Blessing and Ootsuka, 2016; Bourguignon and Storch, 2017). The VTA sends projections to the anterior, lateral, and posterior hypothalamus (Aransay et al., 2015), providing a means for broad communication of ultradian signaling. Inputs to the VTA likely act to fine-tune dopaminergic ultradian activity. For example, pro-opiomelanocortin (POMC) (Qu et al., 2020) neurons of the ARC send projections to the VTA that inhibit dopaminergic activity (Gumbs et al., 2019), and these cells are impacted by kisspeptin (Fu and van den Pol, 2010), providing a mechanism for altering POMC cell input to the VTA based on reproductive/metabolic state. Likewise, agouti-related peptide/neuropeptide Y (AgRP/NPY) neurons project to the VTA, express the *Kiss1r*, and oppose the actions of POMC neuron communication (Kim et al., 2010; Gumbs et al., 2019; Vohra et al., 2022). Finally, the nucleus accumbens (NAcc), a major target of VTA dopamine, projects to both the VTA (Qi et al., 2022) and ARC, potentially to reinforce their coupling (reviewed in (Prendergast and Zucker, 2016)). Together, dopaminergic URs may be an important reinforcer of reproductive-thermoregulatory coupling and have direct neural substrate for communicating with reproductively relevant hypothalamic circuits. However, an additional dopaminergic population within the ARC, tuberoinfundibular dopaminergic (TIDA) neurons, likely further contributes to the integration of reproductive, thermoregulatory, and ovulatory-phase-dependent URs.

TIDA neurons are known to play an important role in reproduction by releasing dopamine into the median eminence to inhibit prolactin secretion (Lyons and Broberger, 2014), with SCN VIP-ergic projections targeting TIDA neurons to time their activity (Freeman et al., 2000), but they may also contribute to the broader pulsatile network. KNDy neurons contact TIDA cells (Sawai et al., 2014; Ozawa, 2021) and, consistent with this connectivity, intracerebroventricular injections of kisspeptin increase prolactin secretion (through inhibition of dopamine release) in an estrogen- and progesterone-dependent manner (Aquino et al., 2019). Moreover, dopaminergic fibers closely appose GnRH fibers at the median eminence (Jennes et al., 1983; Mitchell et al., 2003). In addition, in anestrous ewes, dopamine appears to inhibit GnRH by suppressing KNDy neuron activity via D2 receptors (Goodman et al., 2012; Weems et al., 2017). More specifically, E2 increases expression of D2R, and D2R antagonist infusion in the ARC increases LH pulse frequency (Goodman et al., 2012; Weems et al., 2017). Thus, TIDA neurons may both receive pulsatile input and contribute to the pattern of KNDy and GnRH cell activity (Jennes et al., 1983; Mitchell et al., 2003; Goodman et al., 2012; Sawai et al., 2014; Weems et al., 2017). Dopamine release occurs at intervals ranging from 100 m in to hours, consistent with a role in the ultradian regulation of prolactin (Romanò et al., 2017). Finally, in women, 70% of prolactin (which acts to stimulate TIDA DA release (Moore et al., 1980) pulses occur coincident (within 15 min) with LH pulses, and kisspeptin may act directly on a proportion of TIDA neurons (Aquino et al., 2019), supporting coupled timing of dopamine and kisspeptin pulsatility (Backstrom et al., 1982). These findings point to a putative mechanism whereby dopaminergic output, potentially from the ARC, may reinforce synchronized URs within KNDy, GnRH, LH, and prolactin cells. Together, dopamine from TIDA neurons may synergize with that of the VTA to reinforce coupling among pulses of reproductive hormones and body temperature, as well as potentially contribute to modulation of UR period across the ovulatory cycle.

The mechanisms underlying stereotyped modulation of UR periodicity across the ovulatory cycle are as yet unexplained, but Prendergast and Zucker proposed that changes in dopaminergic tone across the ovulatory cycle could be responsible (Prendergast and Zucker, 2016). If so, one would expect higher dopaminergic tone to be coincident with times of body temperature UR period lengthening, and hormonal output, as well as elevated temperature levels (e.g., the post-ovulatory luteal phase). Likewise, one would expect reduced dopaminergic tone to be associated with increasing frequency of these URs (i.e., the phenotype observed in the pre-ovulatory follicular phase). Consistent with these predictions, prolactin is elevated around ovulation (when UR frequency is high and dopaminergic tone likely reduced), and reduced during the early follicular phase (when UR frequency is low and dopaminergic tone is likely increased) (Franchimont et al., 1976). Moreover, under natural conditions, increases in dopaminergic tone are inversely proportional to increases in LH pulse frequency, potentially from either the VTA or TIDA neurons. Together, recent work reinforces the intriguing possibility that the DUO (Blum et al., 2014) and the ARC hypotheses of ultradian rhythm generation (Prendergast and Zucker, 2016) could be united by direct or indirect communication among the ARC pulse generator, VTA, and TIDA neuron populations (Prendergast and Zucker, 2016).

### Perturbation of coupling

As described herein, central reproductive and thermoregulatory circuits are coupled within the hypothalamus and dopaminergic circuits within the hypothalamus, VTA, and NAcc may enable modulation of ultradian frequency of temperature and reproductive output across the ovulatory cycle. However, numerous other factors contribute to thermoregulation that may perturb this harmony. For example, brown adipose tissue deposition is positively correlated with progesterone secretion across the cycle, inversely correlated with estrogen during the follicular phase, and is heavily influenced by cortisol (Baker et al., 2020; Fuller-Jackson et al., 2020). Unlike the HPG axis, URs in the hypothalamic-pituitary-adrenal axis can be preserved in the absence of URs in their hypothalamic releasing hormone, corticotropin-releasing hormone (Walker et al., 2012). As HPA axis activity contributes to the regulation of body temperature (Hampl et al., 2006; Ramage et al., 2016), and has been observed in limited cases to time-lock with ultradian rhythms of CBT (Smarr et al., 2016a), central reproductive-thermoregulatory coupling is not likely to generate an isolated, 1:1 relationship between these systems, but may mask the relationship between reproductive status and temperature oscillations. The impacts of stress, environment, and behavior on thermoregulation and reproduction have been reviewed recently (McMurray and Katz, 1990; Charkoudian and Stachenfeld, 2011; Morrison and Nakamura, 2011), and lend insight into the complexities of understanding and mapping the coupling between these systems.

If peripheral factors could perturb coupling, this dysregulation could lead to false inferences made from temperature about reproductive state, especially in cases of rhythmic instability (e.g., in shift workers, individuals with diabetes, in stress disorders, or during the perimenopausal period) (Boyle et al., 2018; Katulski et al., 2018; Grant et al., 2020). In particular, the unpleasant side effects of rhythmic disruptors, such as shift work, are due to dissociation of rhythmicity among systems in the body. When the brain and body are required to “adjust” to a new time zone, different systems adjust at different rates, leading to decoupling and suboptimal function during the readjustment period, and the associated malaise of jetlag (Evans and Davidson, 2013; Casper and Gladanac, 2014). Although physical time zone shifts are the most well-known example of rhythmic disruption, many common behaviors and medical interventions are associated with such internal desynchrony. These include insufficient light during the day, blue light exposure at night, late meals, and pharmaceuticals such as hormonal contraception, which together impose “pharmaceutical” or “social” jetlag on an alarming proportion of the population (Rutters et al., 2014; Wong et al., 2015; Smarr and Schirmer, 2018; Grant et al., 2021). The result of these other forms of de-coupling events on thermoregulation make it challenging to apply temperature as a reliable proxy for reproductive state (Smarr et al., 2016a; Grant et al., 2020). Thus, it is important to consider temperature-reproductive ultradian rhythms in the context of perturbing events.

## Summary and conclusions

Reproductive and thermoregulatory hypothalamic circuits are coupled, and both have pulsatile output. Multiple hypotheses have been proposed for how such coupling may be achieved, including the ARC pulse generator and KNDy neurons, the DUO, and decentralized mechanisms. We propose that pulsatility is likely to be of central origin in the case of reproductive-thermoregulatory coupling, that peripheral factors likely reinforce this coupling, and that existing theories about the origins of URs may be complementary. Future studies will help to resolve the specific interplay among the DUO and ARC pulse generators and reproductive and thermoregulatory circuits. Dopamine represents a likely substrate for communication among pulse generators in ARC and the VTA. These systems working together may tune ultradian periodicity across the ovulatory cycle through frequency modulation of ultradian rhythms. There is strong evidence to support the development of continuous temperature-based proxies for reproductive system output, but these proxies may be disrupted by desynchronizing behavioral, environmental, or pharmacological interventions, making interpretation and diagnostics challenging. However, by considering and computationally filtering the contribution of these perturbations, continuous temperature monitoring has broad applications in tracking adolescent development, fertility and infertility, pregnancy, and menopause.
